# Molecular detection of *Haemophilus influenzae* type b and non-typeable strains by PCR in infants under one year hospitalized with community-acquired pneumonia in Peru, 2010–2012

**DOI:** 10.1016/j.nmni.2025.101655

**Published:** 2025-10-30

**Authors:** Miguel Angel Aguilar-Luis, Wilmer Silva-Caso, Angela Cornejo-Tapia, Erico Cieza-Mora, Pablo Weilg, Carlos Bada, Olguita del Aguila, Juana del Valle-Mendoza

**Affiliations:** a Biomedicine Laboratory, Research and Innovation Centre of the Faculty of Health Sciences, Universidad Peruana de Ciencias Aplicadas, 15087, Lima, Peru; b Servicio de Pediatría. Hospital Regional de Salud de Cajamarca, Peru; c Servicio de Pediatría. Hospital de Emergencias Pediátricas, Lima, Peru; d Servicio de Pediatría. Hospital Edgardo Rebagliati Martins, Lima, Peru

**Keywords:** *Haemophilus influenzae*, Hib, NTHi, Community-acquired pneumonia, CAP, Infants, Peru, PCR

## Abstract

**Background:**

Community-acquired pneumonia (CAP) is one of the most common causes of morbidity and mortality among children under five worldwide. *Haemophilus influenzae*—particularly encapsulated serotype b (Hib) and non-typeable strains (NTHi)—remains an important pathogen. Peru introduced Hib vaccination nationally in 2004, but pediatric molecular data from the early post-introduction period are limited.

**Objectives:**

To estimate the molecular prevalence of Hib and NTHi and identify associated clinical/epidemiological factors among infants (<1 year) hospitalized with CAP in Peru during 2010–2012, providing an early post-introduction baseline to inform long-term trends. *Method*: We conducted a prospective multicenter study in five hospitals. Nasopharyngeal swabs underwent conventional PCR for *H. influenzae* detection (1000-bp) and serotyping (Hib 310-bp; NTHi 550-bp). Associations were evaluated using χ^2^/Fisher's tests and multivariable logistic/multinomial regression.

**Results:**

Among 339 infants, *H. Influenzae* was detected in 26.8 % (91/339): Hib 24.2 % (22/91), NTHi 3.3 % (3/91), and other encapsulated serotypes 72.5 % (66/91). In adjusted models, absence of documented Hib vaccination before admission was independently associated with Hib detection (p < 0.001). Atelectasis was associated with non-b encapsulated serotypes (RRR 2.41; 95 % CI 1.02–5.74; p = 0.046). Age and sex showed no independent associations.

**Conclusion:**

These findings do not represent the current epidemiology; rather, they delineate an early post-introduction baseline for Hib/NTHi in Peruvian infants with CAP. Our findings contribute to the timeline of *H. influenzae* epidemiology in Peru, supports evaluations of vaccine impact over time, and underscores the need for sustained molecular surveillance and on-schedule Hib vaccination.

## 1 Introduction

Community-acquired pneumonia (CAP) remains a leading cause of morbidity and mortality in children under five worldwide [1,2]. Among bacterial etiologies *Haemophilus influenzae* (Hi) – notably encapsulated serotype b (Hib) and non-typeable strains (NTHi)- continues to play an important role despite decades of Hib conjugate vaccine use [3,4]. Encapsulated serotypes are classically linked to invasive pediatric infections such as meningitis, septicemia, and pneumonia, whereas NTHi predominates in mucosal infections but has increasingly been implicated in severe disease among vulnerable groups [4,5].

Peru introduced Hib vaccination nationwide in 2004, with infant doses incorporated into the routine Schedule [6]. Subsequent surveillance in Latin America has reported generally high national coverage but also heterogeneity across regions and periods, including disruptions during the COVID-19 pandemic [7], [8], [9], [10], [11]. In parallel, countries with sustained Hib programs have reported epidemiologic shifts: a relative decline in Hib and, in some settings, greater prominence of NTHi and non–type b encapsulated serotypes, alongside growing concerns about antimicrobial resistance [4,5,12]. These dynamics underscore the value of historical baselines to interpret trends over time.

In Peru, the geographic and climatic diversity from high-altitude Andean regions to colder southern zones, shapes respiratory seasonality and access to timely care, factors that can modulate observed CAP patterns and vaccine program performance [2,13]. Programmatic updates such as adoption of combination/hexavalent vaccines aim to sustain coverage and simplify logistics, while equity-oriented strategies target subnational gaps that persisted through and beyond the pandemic period [6,9]. Understanding Hi epidemiology in early infancy is particularly relevant because the window before the first Hib dose leaves neonates and young infants disproportionately vulnerable, even when overall coverage is high.

Establishing *H. influenzae* as an etiologic agent of CAP in infants is methodologically challenging. Culture and agglutination show limited sensitivity and specificity for Hi identification and capsule typing, while upper respiratory tract (URT) detection alone does not establish lower respiratory tract causation and co-pathogens are common [[14], [15], [16]]. Molecular tools, particularly PCR-based detection and capsule-typing targets, can improve identification and serotype attribution [[17], [18], [19]]. However, Hi PCR-based data in Peruvian infants hospitalized with CAP remain scarce, as most regional reports emphasize invasive disease surveillance or carriage in other populations [20], [21], [22], [23]. In addition, interactions with other pediatric vaccine-preventable pathogens (e.g., pneumococcus) and health service disruptions can influence the relative contribution of Hi to severe respiratory disease, reinforcing the importance of temporally anchored datasets o contextualize trends [3,9,15].

To inform the epidemiologic timeline of H. influenzae in Peru, we determined the molecular prevalence of Hib and NTHi and evaluated associated clinical and epidemiological factors among infants (<1 year) hospitalized with CAP during 2010–2012. We explicitly define this cohort as an early post-introduction baseline, rather than as a representation of current epidemiology, situated a few years after the national Hib vaccine rollout [6]. This baseline complements subsequent surveillance and program updates, enabling comparisons across periods to assess vaccine impact and evolving serotype distributions.

To inform Peru's epidemiologic timeline for *H. influenzae*, we estimated the molecular prevalence of Hib and NTHi and evaluated associated clinical and epidemiological factors among infants (<1 year) hospitalized with CAP during 2010–2012. We explicitly frame this cohort as an early post-introduction baseline, a few years after nationwide Hib rollout, to enable comparisons with later surveillance and program updates.

## 2 Material and methods

### 2.1 Study design and setting

We conducted a prospective, multicenter observational study from January 2010 to December 2012 in five public tertiary hospitals in Peru: Hospital Nacional Cayetano Heredia (HNCH), Hospital de Emergencias Pediatricas (HEP), Hospital Nacional Edgardo Rebagliati Martins (HNERM) and Instituto Nacional de Salud del Niño (INSN) in Lima, and Hospital Regional de Cajamarca (HRDC) in Cajamarca.

Clinical diagnosis of pneumonia was made by physicians who were directly involved in the patient's care and was based on physical examination evidence of an acute infectious process or radiologic evidence of an acute pulmonary infiltrate, as well as signs and symptoms of respiratory distress. Vaccination against Hib was introduced nationwide in Peru in 2004; therefore, our study period represents an early post-introduction baseline.

### 2.2 Participants

The study enrolled infants aged 12 months or younger who were hospitalized with community-acquired pneumonia (CAP). Eligibility was assessed consecutively at each site by study clinicians.

### 2.3 Case definition

CAP was diagnosed by the attending physician based on (i) acute respiratory symptoms/signs (e.g., cough, tachypnea, retractions, nasal flaring, grunting) with supportive lung findings on examination and/or (ii) radiographic evidence of acute pulmonary infiltrate. For neonates/young infants, feeding difficulty, irritability, or poor activity in lieu of classic respiratory signs were considered suggestive. In the absence of tachypnea, a chest X-ray was unnecessary; moderate–severe respiratory distress was more common than tachypnea, so this scenario was uncommon. Patients with previous lung conditions or immunodeficiencies were excluded since these conditions are associated with altered microbiota in the upper respiratory tract (URT) and a higher baseline probability of *H. influenzae* carriage compared with otherwise healthy infant.

### 2.4 Data sources and variables

Demographic, clinical, radiographic, and epidemiological data were abstracted from standardized hospital records using a pre-piloted form. Immunization status (Hib/pentavalent doses) was verified by immunization cards or a structured caregiver interview. Variables included age group, sex, nutritional status, prior hospitalization, ICU admission, daycare attendance, household contacts with respiratory symptoms, hospital (site) and radiographic findings (including atelectasis).

During the study period (2010–2012), Peru's National Immunization Program delivered Hib as part of the pentavalent vaccine (DTP–HepB–Hib) given at 2, 4, and 6 months of age in the public sector, free of charge and recorded on a standardized immunization card. No routine Hib booster was included in the infant schedule at that time.

### 2.5 Sample collection

Nasopharyngeal samples were collected by gently inserting a Mini-Tip Culture Direct swab (Becton-Dickinson Microbiology System, USA) into each nostril, parallel to the palate. Both swabs were then combined in a single tube with 2 mL of 1 × PBS and transported to the site laboratory under routine biosafety procedures.

### 2.6 DNA extraction

DNA was extracted from a volume of 200 μL of each sample using a commercial kit (High Pure Template Preparation Kit, Roche Applied Science, Germany) following the manufacturer's protocol. The extracted DNA was stored at −20 °C until molecular analysis [17].

### 2.7 PCR detection and serotyping

*H*. *influenzae* was detected by conventional PCR using Hi-Forward 5′-ATA ACA ACG AAG GGA CTA ACG-3′ and Hi-Reverse 5′-ACC TAC ACC CAC TGA TTT TTC-3′, (expected amplicon ≈1000 bp) [19]. NTHi was identified using NTHi–Forward 5′-TTG CTT CAA CTG ACG AGA AG-3′ and NTHi–Reverse 5′-TTG TCG GCA ACA ACT GGA G-3 (550 bp); Hib-Forward 5′-GGC GAA ATG GTG CTG GTA A -3′ and Hib-Reverse 5′- AAC TCA ACC GAA AGT GAG AGA -3′ (310 bp) [17].

PCR amplification reactions were performed in a final volume of 20 μL using KAPA Taq ReadyMix (Cat. # KK1006, KAPA Biosystems, South Africa). Thermocycling: initial denaturation 94 °C, 5 min; 45 cycles of 94 °C, 1 min/55 °C, 1 min/72 °C, 1 min; final extension 72 °C, 10 min. Amplicons were resolved on 1.5 % agarose stained with ethidium bromide and visualized under UV.

### 2.8 Statistical analysis

Analyses were performed in Stata 18.0 BE (StataCorp, College Station, TX). Categorical variables were summarized as counts/percentages and compared with χ^2^ or Fisher's exact tests, as appropriate. Logistic regression for any H. influenzae detection (positive vs negative), and Multinomial logistic regression for three-category outcome (Hib/other encapsulated serotypes/negative). Variables with clinical plausibility or p < 0.20 in univariable screening were considered for adjusted models. Adjusted odds ratios (OR) or relative risk ratios (RRR) were reported with corresponding 95 % confidence intervals (CI) and p-values. A two-sided p-value<0.05 indicated statistical significance. For sparse categories (e.g., NTHi), results are descriptive or pooled to preserve model stability.

### 2.9 Ethical considerations

The protocol was approved by the Institutional Ethics Committees of HNERM and Hospital Regional Docente de Cajamarca; site permissions were obtained for HNCH, INSN and HEP. Parents or legal guardians provided written informed consent prior to enrollment. Data were anonymized and securely managed throughout the study.

## 3 Results

### 3.1 Enrollment and baseline characteristics

A total of 339 infants under one year hospitalized with CAP were included in the study. Most cases involved infants aged less than two months (33.3 %, n = 113) and between two to three months old (36.9 %, n = 125), with a predominance of males (56.3 %, n = 191). Detailed demographic and epidemiological characteristics are presented in Table 1.

**Table 1:** Association between epidemiological factors and the presence of *Haemophilus influenzae* by PCR, including serotype distribution in hospitalized infants with community-acquired pneumonia.

Epidemiological factors		Total cases	Hi by PCR		Serotypes Hi by PCR			
		n = 339, %	n = 91 (%)	Chi^2^/Fisher (p-value)	Hib n = 22 (%)	Other (a, c-f) n = 66 (%)	NTHi n = 3 (%)	Chi^2^/Fisher (p-value)
Age (months)	<2	113 (33.3)	23 (25.3)	**0.022**	6 (27.3)	16 (24.2)	1 (33.3)	0.052
	2–3	125 (36.9)	30 (33.0)		4 (18.2)	24 (36.4)	2 (66.7)	
	4–5	58 (17.1)	20 (22.0)		7 (31.8)	13 (19.7)	0 (0.0)	
	6–11	43 (12.7)	18 (19.8)		5 (22.7)	13 (19.7)	0 (0.0)	
Gender	women	148 (43.7)	43 (47.3)	0.419	12 (54.5)	30 (45.5)	1 (33.3)	0.681
	male	191 (56.3)	48 (52.7)		10 (45.5)	36 (54.5)	2 (66.7)	
Hib vaccination	no	236 (69.6)	55 (60.4)	**0.026**	13 (59.1)	39 (59.1)	3 (100.0)	0.067
	yes	103 (30.4)	36 (39.6)		9 (40.9)	27 (40.9)	0 (0.0)	
Hib vaccine doses	none	236 (69.6)	55 (60.4)	**0.043**	13 (59.1)	39 (59.1)	3 (100.0)	0.088
	1	65 (19.2)	19 (20.9)		5 (22.7)	14 (21.2)	0 (0.0)	
	2	26 (7.7)	11 (12.1)		4 (18.2)	7 (10.6)	0 (0.0)	
	3	12 (3.5)	6 (6.6)		0 (0.0)	6 (9.1)	0 (0.0)	
Malnutrition	no	144 (42.5)	33 (36.3)	0.161	7 (31.8)	25 (37.9)	1 (33.3)	0.540
	yes	195 (57.5)	58 (63.7)		15 (68.2)	41 (62.1)	2 (66.7)	
Hospitalization	no	39 (11.5)	7 (7.7)	0.183	1 (4.5)	6 (9.1)	0 (0.0)	0.635
	yes	300 (88.5)	84 (92.3)		21 (95.5)	60 (90.9)	3 (100.0)	
ICU Admission	no	309 (91.2)	79 (86.8)	0.089	20 (90.9)	57 (86.4)	2 (66.7)	0.115
	yes	30 (8.8)	12 (13.2)		2 (9.1)	9 (13.6)	1 (33.3)	
Atelectasis	no	304 (89.7)	76 (83.5)	**0.024**	20 (90.9)	53 (80.3)	3 (100.0)	0.057
	yes	35 (10.3)	15 (16.5)		2 (9.1)	13 (19.7)	0 (0.0)	
Seizures	no	336 (99.1)	89 (97.8)	0.177	21 (95.5)	65 (98.5)	3 (100.0)	0.108
	yes	3 (0.9)	2 (2.2)		1 (4.5)	1 (1.5)	0 (0.0)	
Mother (close contact)	no	279 (82.3)	70 (76.9)	0.116	19 (86.4)	50 (75.8)	1 (33.3)	0.063
	yes	60 (17.7)	21 (23.1)		3 (13.6)	16 (24.2)	2 (66.7)	
Father (close contact)	no	320 (94.4)	84 (92.3)	0.311	22 (100.0)	59 (89.4)	3 (100.0)	0.224
	yes	19 (5.6)	7 (7.7)		0 (0.0)	7 (10.6)	0 (0.0)	
Siblings <7 years old	no	280 (82.6)	73 (80.2)	0.485	14 (63.6)	57 (86.4)	2 (66.7)	0.070
	yes	59 (17.4)	18 (19.8)		8 (36.4)	9 (13.6)	1 (33.3)	
Siblings 7–10 years old	no	325 (95.9)	86 (94.5)	0.444	21 (95.5)	62 (93.9)	3 (100.0)	0.594
	yes	14 (4.1)	5 (5.5)		1 (4.5)	4 (6.1)	0 (0.0)	
Siblings >10 years old	no	326 (96.2)	90 (98.9)	0.112	22 (100)	65 (98.5)	3 (100.0)	0.506
	yes	13 (3.8)	1 (1.1)		0 (0.0)	1 (1.5)	0 (0.0)	
Uncles/Aunts (close contact)	no	305 (90.0)	83 (91.2)	0.646	21 (95.5)	59 (89.4)	3 (100.0)	0.895
	yes	34 (10.0)	8 (8.8)		1 (4.5)	7 (10.6)	0 (0.0)	
Other epidemiological contacts	no	301 (89.1)	83 (91.2)	0.441	21 (95.5)	59 (89.4)	3 (100.0)	0.797
	yes	37 (10.9)	8 (8.8)		1 (4.5)	7 (10.6)	0 (0.0)	
Daycare attendance	no	333 (98.2)	88 (96.7)	0.197	21 (95.5)	64 (97.0)	3 (100.0)	0.247
	yes	6 (1.8)	3 (3.3)		1 (4.5)	2 (3.0)	0 (0.0)	
Hospital (site)	HNCH (Lima)	67 (19.8)	9 (9.9)	**0.000**	1 (4.5)	8 (12.1)	0 (0.0)	**0.000**
	HEP (Lima)	47 (13.9)	23 (25.3)		10 (45.5)	12 (18.2)	1 (33.3)	
	HNERM (Lima)	82 (24.2)	27 (29.7)		4 (18.2)	23 (34.8)	0 (0.0)	
	INSN (Lima)	60 (17.7)	17 (18.7)		5 (22.7)	11 (16.7)	1 (33.3)	
	HRDC (Cajamarca)	83 (24.5)	15 (16.5)		2 (9.1)	12 (18.2)	1 (33.3)	

Hi: *H. influenzae,* Hib: *H. influenzae* Type b), Other encapsulated types (serotypes a, c, d, e, f), NTHi: *H. influenzae* non-typeable, unencapsulated. Statistically significant results (*p* < 0.05) are highlighted in bold.

### 3.2 PCR detection and serotype distribution

*H*. *influenzae* was detected in 91/339 (26.8 %). Among positives, Hib accounted for 22/91 (24.2 %), NTHi for 3/91 (3.3 %), and other encapsulated serotypes (a, c–f) for 66/91 (72.5 %) (Table 1). Hib was most frequent at 4–5 months (31.8 %), whereas other encapsulated serotypes predominated at 2–3 months (36.4 %) (Table 1).

### 3.3 Bivariable associations

Overall *H. influenzae* detection varied by age group (p = 0.022) with the highest positivity at 2–3 months (33.0 %). Detection did not differ by sex (p = 0.419). Hib vaccination (yes/no) (p = 0.026) and dose count (p = 0.043) correlated with detection; atelectasis was more frequent among *H. influenzae* positive infants (p = 0.024). There was a significant difference in *H. influenzae* detection by hospital (site), ranging from 9.9 % at HNCH to 29.7 % at HNERM and 25.3 % at HEP. Hib detections were most frequent at HEP (45.5 %) among Hi-positive infants, whereas HRDC showed lower overall Hi (16.5 %) (Table 1).

Regarding symptoms, cough (86.1 %), tachypnea (72.9 %), and cyanosis (57.2 %) were common. Cyanosis was associated with overall *H. influenzae* detection at the bivariable level (p = 0.025). Although cough was the most prevalent symptom, it did not show a statistically significant difference when comparing *H. influenzae* positive and negative groups (p = 0.200). Other clinical symptoms such as fever, tachypnea, difficulty feeding, apnea, inspiratory stridor, vomiting after cough, and diarrhea did not show statistically significant associations with Hib or other serotypes (Table 2).

**Table 2:** Relationship between clinical symptoms and *Haemophilus influenzae* detection by PCR, including serotype distribution in hospitalized infants with community-acquired pneumonia.

Clinical symptoms	Total cases	Hi by PCR		Serotypes Hi by PCR			
	n = 339, %	n = 91 (%)	Chi^2^/Fisher (p-value)	Hib n = 22 (%)	Other (a, c-f) n = 66 (%)	NTHi n = 3 (%)	Chi^2^/Fisher (p-value)
Fever			0.334				0.626
no	208 (61.4)	52 (57.1)		13 (59.1)	38 (57.6)	1 (33.3)	
yes	131 (38.6)	39 (42.9)		9 (40.9)	28 (42.4)	2 (66.7)	
Cough			0.200				0.310
no	47 (13.9)	9 (9.9)		3 (13.6)	5 (7.6)	1 (33.3)	
yes	292 (86.1)	82 (90.1)		19 (86.4)	61 (92.4)	2 (66.7)	
Cyanosis			**0.025**				**0.025**
no	145 (42.8)	48 (52.7)		9 (40.9)	36 (54.5)	3 (100.0)	
yes	194 (57.2)	43 (47.3)		13 (59.1)	30 (45.5)	0 (0.0)	
Tachypnea			0.308				0.638
no	92 (27.1)	21 (23.1)		4 (18.2)	17 (25.8)	0 (0.0)	
yes	247 (72.9)	70 (76.9)		18 (81.8)	49 (74.2)	3 (100.0)	
Difficulty feeding			0.938				0.985
no	180 (53.1)	48 (52.7)		11 (50.0)	35 (53.0)	2 (66.7)	
yes	159 (46.9)	43 (47.3)		11 (50.0)	31 (47.0)	1 (33.3)	
Apnea			0.921				0.197
no	282 (83.2)	76 (83.5)		15 (68.2)	58 (87.9)	3 (100.0)	
yes	57 (16.8)	15 (16.5)		7 (31.8)	8 (12.1)	0 (0.0)	
Inspiratory stridor			0.330				0.186
no	262 (77.3)	67 (73.6)		13 (59.1)	51 (77.3)	3 (100.0)	
yes	77 (22.7)	24 (26.4)		9 (40.9)	15 (22.7)	0 (0.0)	
Vomiting after cough			0.178				0.503
no	188 (55.5)	45 (49.5)		10 (45.5)	33 (50.0)	2 (66.7)	
yes	151 (44.5)	46 (50.5)		12 (54.5)	33 (50.0)	1 (33.3)	
Diarrhea			0.202				0.584
no	305 (90.0)	85 (93.4)		21 (95.5)	61 (92.4)	3 (100.0)	
yes	34 (10.0)	6 (6.6)		1 (4.5)	5 (7.6)	0 (0.0)	

Hi: *H. influenzae,* Hib: *H. influenzae* Type b), Other encapsulated types (serotypes a, c, d, e, f), NTHi: *H. influenzae* non-typeable, unencapsulated. Statistically significant results are those with p-value <0.05 and are highlighted in bold.

### 3.4 Multivariable models

Multivariable logistic and multinomial regression analyses identified independent factors associated with *H. influenzae* and its specific serotypes (Hib and other encapsulated types). Absence of Hib vaccination showed a strong significant association exclusively with Hib detection (p < 0.001), consistent with vaccine effectiveness against Hib disease (Table 3). Atelectasis remained independently associated with non-b encapsulated serotypes (RRR = 2.41; 95 % CI: 1.02–5.74; p = 0.046). After controlling for covariates and setting HNCH as the reference site, enrollment at HEP showed higher odds of any *H. influenzae* (OR 2.82; 95 % CI 1.21–6.57; p = 0.016) and a markedly higher relative risk of Hib versus negative (RRR 8.57; 95 % CI 1.81–40.60; p = 0.007), whereas HRDC showed lower odds of overall *H. influenzae* (OR 0.29; 95 % CI 0.09–0.97; p = 0.045). Associations for HNERM and INSN were not statistically significant. Age and sex were not independently associated. Cyanosis did not retain an independent association in adjusted models. The NTHi category was excluded from multinomial contrasts because of low counts and convergence issues; corresponding estimates were unstable (Table 3).

**Table 3:** Multivariable analysis of factors associated with *Haemophilus influenzae* detection and serotypes using logistic and multinomial regression.

Variables	Hi by PCR	Serotypes Hi	
	Hi vs Negative	Hib vs Negative	Other vs Negative
	OR (95 % CI, p-value)	RRR (95 % CI, p-value)	RRR (95 % CI, p-value)
**Epidemiological factors**			
Age (Ref: <2 months)			
2–3	1.18 (0.59–2.34, 0.646)	0.46 (0.11–1.96, 0.293)	1.40 (0.64–3.05, 0.398)
4–5	2.08 (0.87–5.01, 0.101)	2.45 (0.55–10.93, 0.241)	1.79 (0.66–4.87, 0.256)
6–11	2.23 (0.79–6.27, 0.128)	3.56 (0.70–18.04, 0.126)	1.65 (0.48–5.63, 0.423)
Hib vaccination (yes)	1.36 (0.30–6.12, 0.692)	**7.99e-10 (2.06e-10**–**3.11e-09,** **0.000)**	2.70 (0.52–13.97, 0.237)
Hib vaccine Doses (Ref: none)			
1 dose	0.79 (0.17–3.79, 0.773)	**1.88e+09 (4.38e+08 - 1.45e+10, 0.000)**	0.41 (0.08–2.25, 0.306)
2 doses	0.98 (0.21–4.63, 0.978)	**2.52e+09 (4.38e+08 - 1.45e+10,0.000)**	0.53 (0.10–2.81, 0.454)
Malnutrition (yes)	1.28 (0.70–2.33, 0.421)	1.50 (0.47–4.80, 0.491)	1.20 (0.61–2.36, 0.594)
Hospitalization (yes)	1.26 (0.44–3.58, 0.662)	3.88 (0.36–41.96, 0.263)	0.81 (0.26–2.52, 0.713)
ICU Admission (yes)	1.49 (0.60–3.75, 0.391)	0.76 (0.13–4.64, 0.770)	1.67 (0.60–4.69, 0.330)
Atelectasis (yes)	1.91 (0.84–4.34, 0.121)	0.77 (0.11–5.66, 0.788)	**2.41 (1.02**–**5.74, 0.046)**
Seizures (yes)	5.52 (0.38–79.66, 0.269)	7.30 (0.25–215.45, 0.250)	5.47 (0.25–119.80, 0.281)
Mother (close contact) (yes)	1.32 (0.68–2.58, 0.417)	0.48 (0.11–2.10, 0.329)	1.48 (0.72–3.10, 0.304)
Daycare attendance (yes)	2.16 (0.35–13.41, 0.407)	2.16 (0.21–76.08, 0.355)	2.18 (0.30–16.02, 0.443)
Hospital (site) (Ref: HNCH)			
HEP	2.82 (1.21–6.57**, 0.016**)	8.57 (1.81–40.60, **0.007**)	1.65 (0.63–4.35, 0.308)
HNERM	0.89 (0.37–2.11, 0.790)	1.03 (0.17–6.26, 0.971)	0.73 (0.28–1.93, 0.531)
INSN	0.66 (0.28–1.52, 0.326)	0.35 (0.05–2.75, 0.321)	0.69 (0.28–1.71, 0.429)
HRDC	0.29 (0.09–0.97, **0.045**)	0.14 0.00–4.22, 0.255)	0.33 (0.09–1.18, 0.089)
**Clinical symptoms**			
Cough (yes)	1.25 (0.54–2.89, 0.602)	0.89 (0.20–3.90, 0.878)	1.75 (0.62–4.94, 0.291)
Cyanosis (yes)	0.59 (0.33–1.03, 0.062)	0.66 (0.22–1.96, 0.458)	0.61 (0.33–1.14, 0.124)
Apnea (yes)	1.17 (0.53–2.61, 0.697)	1.17 (0.13–5.23, 0.716)	0.74 (0.28–1.96, 0.557)
Vomiting after cough (yes)	1.52 (0.88–2.62, 0.131)	1.76 (0.63–4.91, 0.276)	1.43 (0.78–2.63, 0.246)
Diarrhea (yes)	0.43 (0.16–1.15, 0.094)	1.18 (0.02–1.71, 0.287)	0.54 (0.18–1.58, 0.258)

Results of the multivariable logistic regression for *H. influenzae* detection by PCR and the multinomial logistic regression for serotype classification (Hib and other encapsulated types vs. negative cases). NTHi vs. Negative excluded due to convergence issues and unstable estimates. Note: The category “3 doses of Hib vaccine” was omitted due to collinearity. Statistically significant results (*p* < 0.05) are highlighted in bold.

### 3.5 Vaccination by age group

**Fig. 1:**
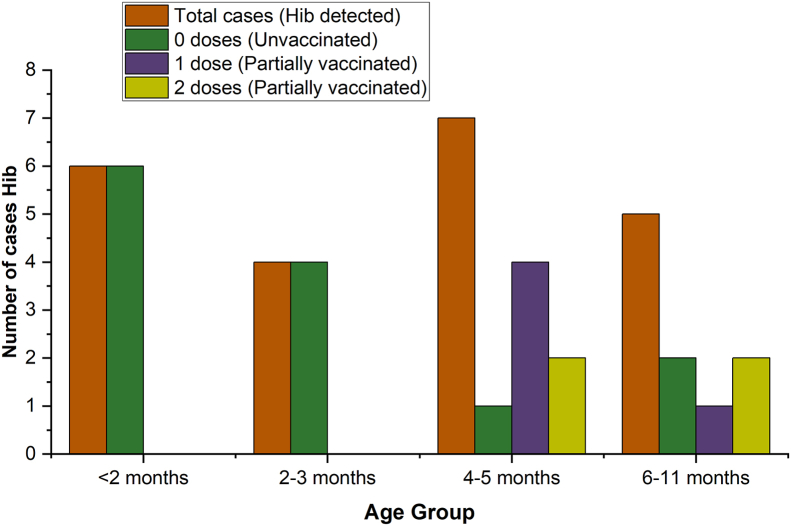
Distribution of Hib cases by age group and vaccination status. Distribution of *Haemophilus influenzae* type b (Hib) cases among infants under one year old hospitalized with community-acquired pneumonia (CAP) in Peru. The data are stratified by age group and vaccination status, highlighting the proportion of Hib-positive cases among vaccinated and non-vaccinated infants.

Among Hib-positive cases, on-schedule coverage was low: infants <2 months (ineligible) had no doses; at 2–3 months, none had received a first dose; at 4–5 months, two had two doses and four had one dose; at 6–11 months, one had completed three doses. Fig. 1 summarizes the age–vaccination distribution.

## 4 Discussion

In this multicenter cohort of Peruvian infants hospitalized with CAP during 2010–2012, *H*. *influenzae* (Hi) was detected by PCR in approximately one quarter of participants, with Hib comprising about one-fourth of detections and non-b encapsulated serotypes the majority of the remainder. Absence of Hib vaccination was strongly associated with Hib detection in adjusted models, while atelectasis showed an association with non-b encapsulated infections. In contrast, cyanosis, although common, was not independently associated with Hi or any serotype after adjustment and should not be interpreted as an etiologic discriminator in this setting.

Rather than a snapshot of current epidemiology, these data represent an early post-introduction baseline for Hi among Peruvian infants with CAP - several years after the nationwide rollout of Hib (2004). International and regional literature describes post-Hib shifts with declining Hib and, in some settings, a larger share of NTHi and non-b encapsulated disease, alongside evolving antimicrobial resistance concerns [[3], [4], [5],^12^]. The coverage in Peru and Latin America has been heterogeneous, including disruptions during COVID-19 [6,7,9]. Against this backdrop, our results provide a chronologic anchor for comparing subsequent surveillance and serotype dynamics across later years.

PCR-based detection and capsule typing address known limitations of culture/agglutination and improve serotype attribution [[17], [18], [19]]. It is important to note, however, that URT detection does not establish lower-respiratory tract causation, and pediatric ARI is likely to involve co-pathogens as well [14,16]. Our multivariable findings should therefore be interpreted as associations rather than causal evidence. The high frequency of cough reported in other pediatric ARI series was not specific for Hib or Hi, reinforcing the limited value of isolated clinical signs in discriminating between the two. The above considerations support the complementary role of molecular testing in refining empiric management and strengthening surveillance and enable serotype-resolved monitoring.

The strong inverse association between on-schedule Hib vaccination and Hib detection is consistent with established vaccine effectiveness and underscores the importance of timely completion of the infant series [9,24]. The age-specific patterns we found-especially the signal at 4–5 months, when many infants have not yet completed the series, highlight a window of vulnerability in which delayed or incomplete dosing may heighten risk. Infants under 2 months of age, who are ineligible for the first Hib dose, also contribute to the disease burden [25]. While maternal Hib vaccination is not part of routine policy in Peru, strengthening perinatal and early-infancy services (e.g., on-time initiation, reminder/recall systems, and targeted outreach in under-served areas) remains the most effective approach to mitigate early risk [7,8].

The adjusted association between atelectasis and non-b encapsulated serotypes suggests potential phenotypic heterogeneity among Hi infections [26], [27], [28]. The presence of this signal may be a result of differences in pathogenesis, timing of care-seeking, radiographic interpretation, or unmeasured confounding; it is consistent with reports of an expansion of serotype contributions beyond Hib in the post-vaccine era. Considering the absence of systematic LRT sampling and multiplex testing, and the aggregate categorization of non-b serotypes, this observation should be considered hypothesis-generating and validated by standardized imaging and serotype-resolved typing.

### 4.1 Limitations

Limitations include hospital-based sampling (potential bias), URT-only testing without systematic LRT cultures or multiplex panels (etiologic uncertainty and under-ascertainment of co-infections), aggregate non-b serotyping (no individual serotype resolution), vaccination records and interviews (possible misclassification), and the historical timeframe (2010–2012). NTHi count was small, which limited the ability to perform adjusted analyses and prevented stable estimates for that group. In addition, our findings should apply to hospitalized infants who are otherwise healthy, rather than immunocompromised children or those who have already developed chronic lung disease, who possess distinct URT microbiotas and are more susceptible to *H. influenzae* carriage may differ from the target population.

## 5 Conclusions

The study provides a molecularly defined, early post-Hib vaccine introduction in Peru baseline for H. influenzae carriage in the upper respiratory tract of infants with community-acquired pneumonia relative to their vaccination status. The results demonstrate the importance of maintaining an infant Hib schedule that is completed on time, as well as maintaining molecular, serotype-resolved surveillance to track shifts in Hib, non-b encapsulated serotypes, and NTHi.

Data from this study indicates that molecular diagnostics may be used to refine empiric management, but given URT sampling only, estimates should be interpreted as associations rather than causal attributions of lower-respiratory disease.

## CRediT authorship contribution statement

**Miguel Angel Aguilar-Luis:** Writing – review & editing, Writing – original draft, Visualization, Supervision, Methodology, Investigation, Formal analysis, Conceptualization. **Wilmer Silva-Caso:** Investigation, Conceptualization. **Angela Cornejo-Tapia:** Methodology. **Erico Cieza-Mora:** Investigation, Conceptualization. **Pablo Weilg:** Writing – original draft. **Carlos Bada:** Methodology, Investigation. **Olguita del Aguila:** Methodology, Investigation. **Juana del Valle-Mendoza:** Writing – review & editing, Supervision, Project administration, Funding acquisition, Conceptualization.

## Consent to publish

All authors have given their authorization for the publication of the manuscript.

## Availability of data and materials

Abstraction format used in the study and dataset are available and accessible from corresponding author upon request.

## Ethics approval and consent to participate

This study has been approved by two independent Ethics Committees from *Hospital Nacional Edgardo Rebagliaty Martins* and *Hospital Regional de Cajamarca.* All samples were analyzed after a written informed consent was signed by parents or children's caregivers.

## Funding

This research and the APC was funded by the *Universidad Peruana de Ciencias Aplicadas* (UPC, Grant N° 2017-EXP-5).

## Declaration of competing interest

The authors declare that they have no known competing financial interests or personal relationships that could have appeared to influence the work reported in this paper.

## References

[1] Lv G., Shi L., Liu Y., Sun X., Mu K. (2024). Epidemiological characteristics of common respiratory pathogens in children. Sci Rep.

[2] Padilla J., Espíritu N., Rizo-Patrón E., Medina M.C. (2017). Children pneumonia in Peru: epidemiologic trends, interventions and progress. Revista Médica Clínica Las Condes.

[3] Gilsdorf J.R. (2021). Hib vaccines: their impact on Haemophilus influenzae type b disease. JID (J Infect Dis).

[4] Slack M.P.E., Cripps A.W., Grimwood K., Mackenzie G.A., Ulanova M. (2021). Invasive haemophilus influenzae infections after 3 decades of hib protein conjugate vaccine use. Clin Microbiol Rev.

[5] Zanella R.C., Bokermann S., Galhardo M., Gava C., Almeida S.C.G., Pereira G.A. (2024). Trends in serotype distribution and antimicrobial susceptibility pattern of invasive Haemophilus influenzae isolates from Brazil, 2009–2021. Int Microbiol.

[6] Torres-Martinez C., Chaparro E., Mariño A.C., Falleiros-Arlant L.H., Camacho-Moreno G., Castillo M.E. (2023). Recommendations for modernizing infant vaccination schedules with combination vaccines in Colombia and Peru. Rev Panam Salud Publica.

[7] Mezones-Holguin E., Al-kassab-Córdova A., Maguiña J.L., Rodriguez-Morales A.J. (2021). Vaccination coverage and preventable diseases in Peru: reflections on the first diphtheria case in two decades during the midst of COVID-19 pandemic. Trav Med Infect Dis.

[8] MINSA Tablero de informacion de Inmunizaciones 2018-2024, Peru 2024:1. https://www.minsa.gob.pe/reunis/data/Indicadores_Inmunizaciones.asp.

[9] Silva L.A.N., Costa F.S., Cata-Preta B.O., Huicho L., Lanata C.F., Araujo M.A.M. (2025). Trends in coverage following an equity-oriented strategy for introducing new vaccines, Peru, 2004-2022. Bull World Health Organ.

[10] Gomez J.A., Tirado J.C., Navarro Rojas A.A., Castrejon Alba M.M., Topachevskyi O. (2013). Cost-effectiveness and cost utility analysis of three pneumococcal conjugate vaccines in children of Peru. BMC Public Health.

[11] Brueggemann A.B., Jansen van Rensburg M.J., Shaw D., McCarthy N.D., Jolley K.A., Maiden M.C.J. (2021). Changes in the incidence of invasive disease due to Streptococcus pneumoniae, Haemophilus influenzae, and Neisseria meningitidis during the COVID-19 pandemic in 26 countries and territories in the invasive respiratory infection surveillance initiative: a prospective analysis of surveillance data.

[12] Oliver S.E., Rubis A.B., Soeters H.M., Reingold A., Barnes M., Petit S. (2023). Epidemiology of invasive nontypeable Haemophilus influenzae disease-United States, 2008-2019. Clin Infect Dis.

[13] OPS/OMS (2014).

[14] Hinz R., Zautner A., Hagen R., Frickmann H. (2015). Difficult identification of Haemophilus influenzae, a typical cause of upper respiratory tract infections, in the microbiological diagnostic routine. Eur J Microbiol Immunol.

[15] Slack M., Esposito S., Haas H., Mihalyi A., Nissen M., Mukherjee P. (2020). Haemophilus influenzae type b disease in the era of conjugate vaccines: critical factors for successful eradication.

[16] Wei L., Liu W., Zhang X.A., Liu E.M., Wo Y., Cowling B.J. (2015). Detection of viral and bacterial pathogens in hospitalized children with acute respiratory illnesses, Chongqing, 2009–2013. Medicine.

[17] García M.C., Lozano P., Antonio Rivera J., Giono S., Martínez Y., Del Carmen Rocha-gracia R. (2008). Identificación y tipificación de Haemophilus influenzae mediante PCR múltiple. Universitas Médica.

[18] Reddington K., Schwenk S., Tuite N., Platt G., Davar D., Coughlan H. (2015). Comparison of established diagnostic methodologies and a novel bacterial smpB real-time PCR assay for specific detection of Haemophilus influenzae isolates associated with respiratory tract infections. J Clin Microbiol.

[19] Weltman G., Fossati M., Correa C., Regueira M., Mollerach M. (2005). Tipificación capsular mediante PCR de aislamientos de Haemophilus influenzae no tipificables por aglutinación. Rev Argent Microbiol.

[20] Efron A., Nápoli D., Neyro S., Juárez M. del V., Moscoloni M., Eluchans N.S. (2023). Laboratory surveillance of invasive Haemophilus influenzae disease in Argentina, 2011–2019[Vigilancia epidemiológica por laboratorio de enfermedad invasiva causada por Haemophilus influenzae en Argentina, 2011-2019].

[21] Juárez X., Flores Yavi R., Balboa R., Matteucci E., Burundarena C., Causarano M.F. (2023). Invasive Haemophilus influenzae disease: a report of 14 cases one year after the COVID-19 pandemic outbreak[Enfermedad invasiva por Haemophilus influenzae: reporte de 14 casos luego de un año de iniciada la pandemia por COVID-19].

[22] Zanella R.C., De Cunto Brandileone M.C., Almeida S.C.G., De Lemos A.P.S., Sacchi C.T., Gonçalves C.R. (2019).

[23] Palaniappan P.A., Mohamed Sukur S., Liow Y.L., Maniam S., Sherina F., Ahmad N. (2020). Carriage of Haemophilus influenzae among children attending childcare centres in Kuala Lumpur, Malaysia in the post vaccination era: a cross-sectional study. Vaccine.

[24] Juscamayta-López E., Valdivia F., Soto M.P., Horna H., Pajuelo M. (2023). Case-control study to estimate the association between tdap vaccination during pregnancy and reduced risk of pertussis in newborn infants in Peru, 2019-2021.

[25] Mackenzie G.A., Hill P.C., Jeffries D.J., Ndiaye M., Sahito S.M., Hossain I. (2021). Impact of the introduction of pneumococcal conjugate vaccination on invasive pneumococcal disease and pneumonia in the Gambia: 10 years of population-based surveillance. Lancet Infect Dis.

[26] Huss N.P., Majeed S.T., Wills B.M., Tarakanova V.L., Brockman K.L., Jondle C.N. (2024). Nontypeable Haemophilus influenzae challenge during gammaherpesvirus infection enhances viral reactivation and latency. Virology.

[27] Atto B., Gell D., Tristram S. (2021). Exploiting the struggle for haem: a novel therapeutic approach against Haemophilus influenzae. Microbiol Aust.

[28] Kubota M., Kenri T., Sasaki Y., Shibayama K., Takayanagi K., Kenzaka T. (2020). Complete genome sequence of a Japanese clinical isolate of Haemophilus influenzae type a strain TAMBA230. Microbiol Resour Announc.

